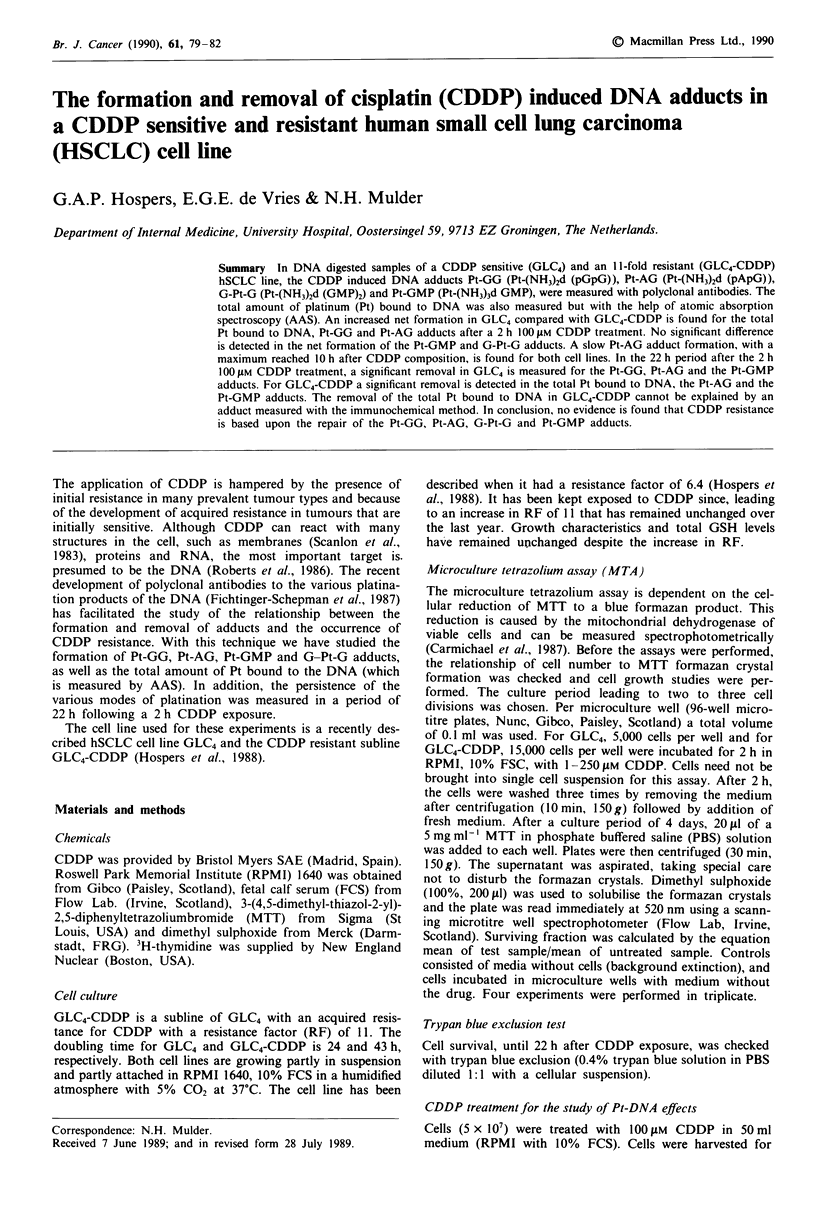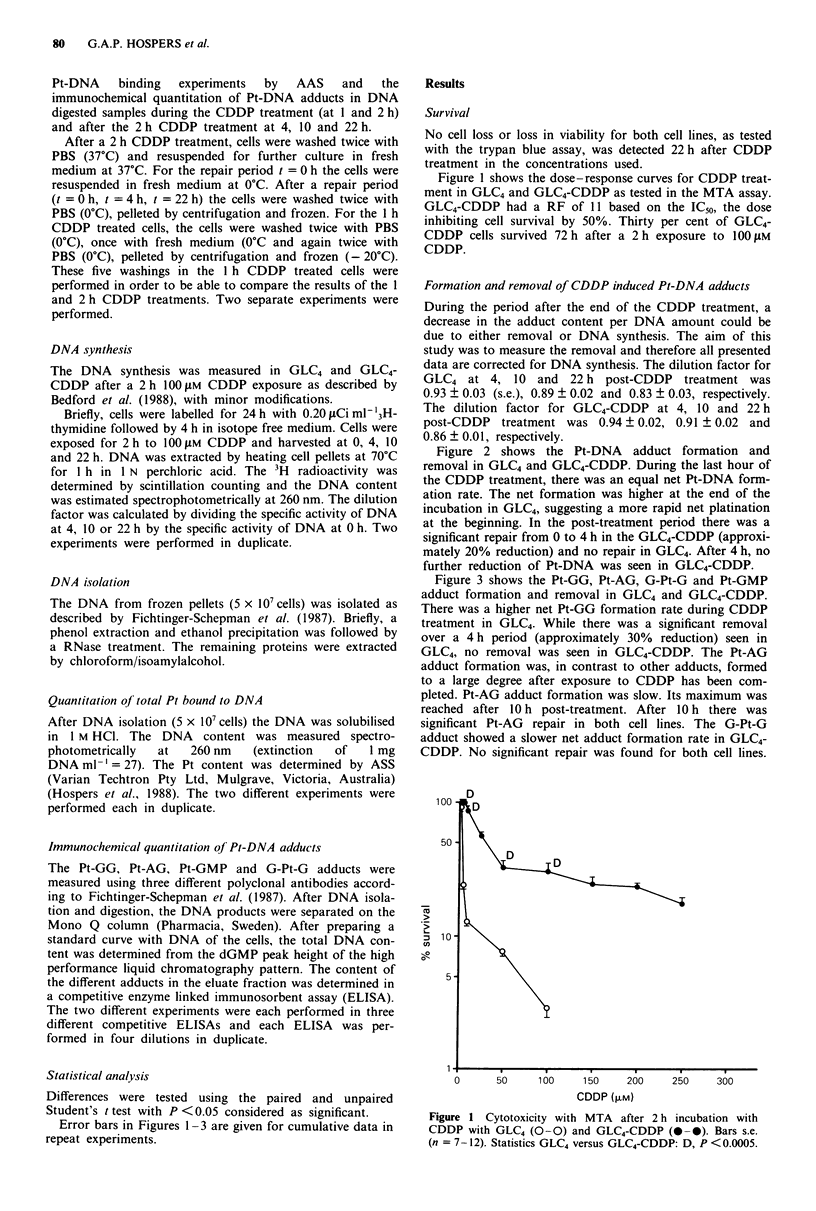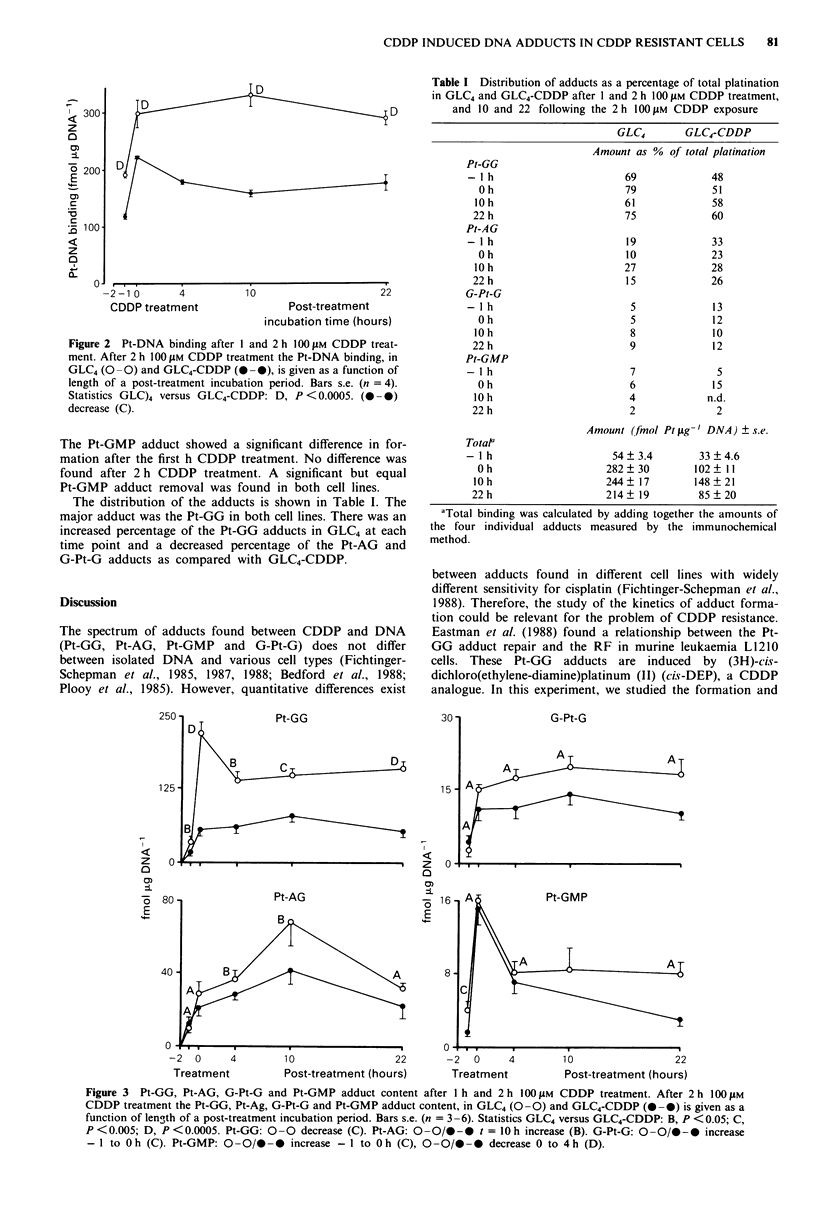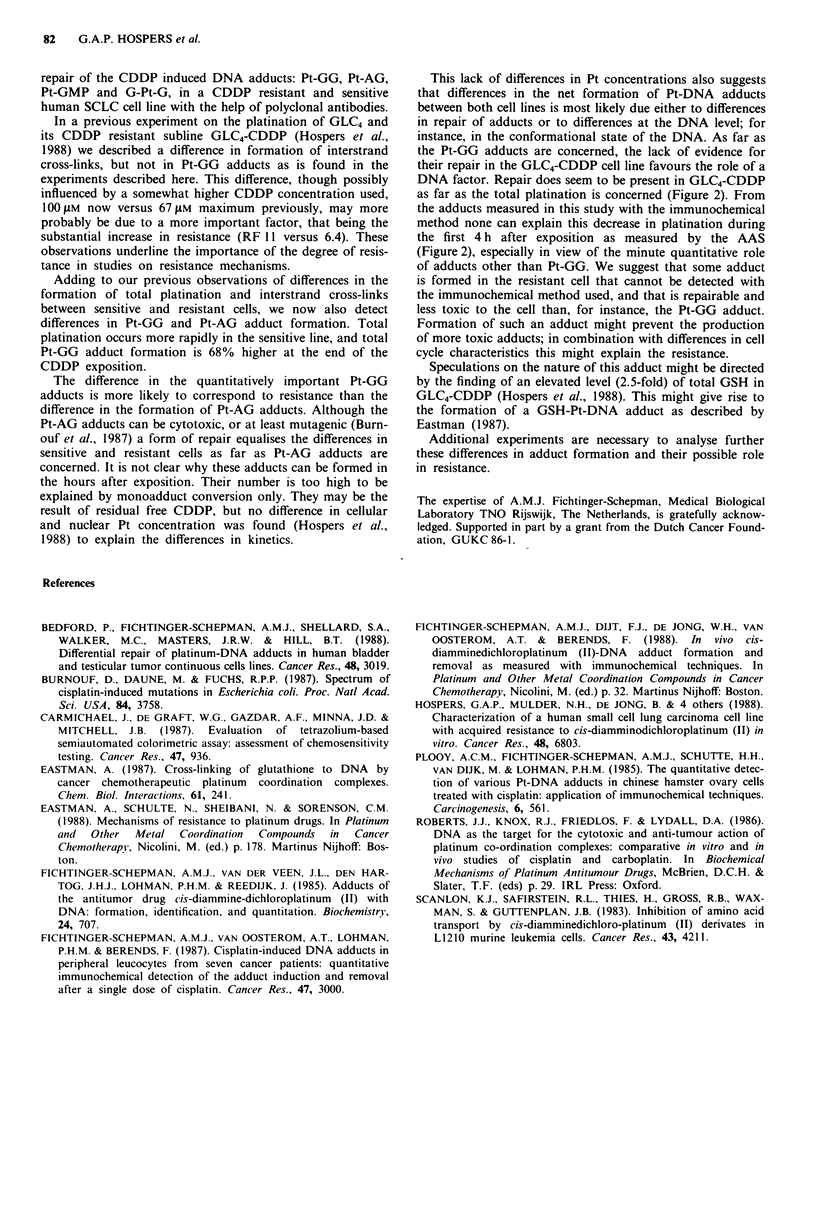# The formation and removal of cisplatin (CDDP) induced DNA adducts in a CDDP sensitive and resistant human small cell lung carcinoma (HSCLC) cell line.

**DOI:** 10.1038/bjc.1990.18

**Published:** 1990-01

**Authors:** G. A. Hospers, E. G. de Vries, N. H. Mulder

**Affiliations:** Department of Internal Medicine, University Hospital, Groningen, The Netherlands.

## Abstract

In DNA digested samples of CDDP sensitive (GLC4) and an 11-fold resistant (GLC4-CDDP) hSCLC line, the CDDP induced DNA adducts Pt-GG (Pt-(NH3)2d (pGpG], Pt-AG (Pt-(NH3)2d (pApG], G-Pt-G (Pt-(NH3)2d (GMP)2) and Pt-GMP (Pt-(NH3)3d GMP), were measured with polyclonal antibodies. The total amount of platinum (Pt) bound to DNA was also measured but with the help of atomic absorption spectroscopy (AAS). An increased net formation in GLC4 compared with GLC4-CDDP is found for the total Pt bound to DNA, Pt-GG and Pt-AG adducts after a 2 h 100 microM CDDP treatment. No significant difference is detected in the net formation of the Pt-GMP and G-Pt-G adducts. A slow Pt-AG adduct formation, with a maximum reached 10 h after CDDP composition, is found for both cell lines. In the 22 h period after the 2 h 100 microM CDDP treatment, a significant removal in GLC4 is measured for the Pt-GG, Pt-AG and the Pt-GMP adducts. For GLC4-CDDP a significant removal is detected in the total Pt bound to DNA, the Pt-AG and the Pt-GMP adducts. The removal of the total Pt bound to DNA in GLC4-CDDP cannot be explained by an adduct measured with the immunochemical method. In conclusion, no evidence is found that CDDP resistance is based upon the repair of the Pt-GG, Pt-AG, G-Pt-G and Pt-GMP adducts.


 © Macmillan Press Ltd., 1990

### The formation and removal of cisplatin (CDDP) induced DNA adducts in a CDDP sensitive and resistant human small cell lung carcinoma (HSCLC) cell line

G.A.P. Hospers, E.G.E. de Vries & N.H. Mulder

*Department of Internal Medicine, University Hospital, Oostersingel 59, 9713 EZ Groningen, The Netherlands.*

**Summary** In DNA digested samples of a CDDP sensitive ($GLC_4$) and an 11-fold resistant ($GLC_4$-CDDP) hSCLC line, the CDDP induced DNA adducts Pt-GG (Pt-$(NH_3)_2$d (pGpG)), Pt-AG (Pt-$(NH_3)_2$d (pApG)), G-Pt-G (Pt-$(NH_3)_2$d $(GMP)_2$) and Pt-GMP (Pt-$(NH_3)_3$d GMP), were measured with polyclonal antibodies. The total amount of platinum (Pt) bound to DNA was also measured but with the help of atomic absorption spectroscopy (AAS). An increased net formation in $GLC_4$ compared with $GLC_4$-CDDP is found for the total Pt bound to DNA, Pt-GG and Pt-AG adducts after a 2 h 100 μM CDDP treatment. No significant difference is detected in the net formation of the Pt-GMP and G-Pt-G adducts. A slow Pt-AG adduct formation, with a maximum reached 10 h after CDDP composition, is found for both cell lines. In the 22 h period after the 2 h 100 μM CDDP treatment, a significant removal in $GLC_4$ is measured for the Pt-GG, Pt-AG and the Pt-GMP adducts. For $GLC_4$-CDDP a significant removal is detected in the total Pt bound to DNA, the Pt-AG and the Pt-GMP adducts. The removal of the total Pt bound to DNA in $GLC_4$-CDDP cannot be explained by an adduct measured with the immunochemical method. In conclusion, no evidence is found that CDDP resistance is based upon the repair of the Pt-GG, Pt-AG, G-Pt-G and Pt-GMP adducts.

The application of CDDP is hampered by the presence of initial resistance in many prevalent tumour types and because of the development of acquired resistance in tumours that are initially sensitive. Although CDDP can react with many structures in the cell, such as membranes (Scanlon *et al.*, 1983), proteins and RNA, the most important target is presumed to be the DNA (Roberts *et al.*, 1986). The recent development of polyclonal antibodies to the various platination products of the DNA (Fichtinger-Schepman *et al.*, 1987) has facilitated the study of the relationship between the formation and removal of adducts and the occurrence of CDDP resistance. With this technique we have studied the formation of Pt-GG, Pt-AG, Pt-GMP and G-Pt-G adducts, as well as the total amount of Pt bound to the DNA (which is measured by AAS). In addition, the persistence of the various modes of platination was measured in a period of 22 h following a 2 h CDDP exposure.

The cell line used for these experiments is a recently described hSCLC cell line $GLC_4$ and the CDDP resistant subline $GLC_4$-CDDP (Hospers *et al.*, 1988).

### Materials and methods

### *Chemicals*

CDDP was provided by Bristol Myers SAE (Madrid, Spain). Roswell Park Memorial Institute (RPMI) 1640 was obtained from Gibco (Paisley, Scotland), fetal calf serum (FCS) from Flow Lab. (Irvine, Scotland), 3-(4,5-dimethyl-thiazol-2-yl)-2,5-diphenyltetrazoliumbromide (MTT) from Sigma (St Louis, USA) and dimethyl sulphoxide from Merck (Darmstadt, FRG). $^3$H-thymidine was supplied by New England Nuclear (Boston, USA).

### *Cell culture*

$GLC_4$-CDDP is a subline of $GLC_4$ with an acquired resistance for CDDP with a resistance factor (RF) of 11. The doubling time for $GLC_4$ and $GLC_4$-CDDP is 24 and 43 h, respectively. Both cell lines are growing partly in suspension and partly attached in RPMI 1640, 10% FCS in a humidified atmosphere with 5% $CO_2$ at 37°C. The cell line has been described when it had a resistance factor of 6.4 (Hospers *et al.*, 1988). It has been kept exposed to CDDP since, leading to an increase in RF of 11 that has remained unchanged over the last year. Growth characteristics and total GSH levels have remained unchanged despite the increase in RF.

### *Microculture tetrazolium assay (MTA)*

The microculture tetrazolium assay is dependent on the cellular reduction of MTT to a blue formazan product. This reduction is caused by the mitochondrial dehydrogenase of viable cells and can be measured spectrophotometrically (Carmichael *et al.*, 1987). Before the assays were performed, the relationship of cell number to MTT formazan crystal formation was checked and cell growth studies were performed. The culture period leading to two to three cell divisions was chosen. Per microculture well (96-well microtitre plates, Nunc, Gibco, Paisley, Scotland) a total volume of 0.1 ml was used. For $GLC_4$, 5,000 cells per well and for $GLC_4$-CDDP, 15,000 cells per well were incubated for 2 h in RPMI, 10% FSC, with 1–250 μM CDDP. Cells need not be brought into single cell suspension for this assay. After 2 h, the cells were washed three times by removing the medium after centrifugation (10 min, 150 *g*) followed by addition of fresh medium. After a culture period of 4 days, 20 μl of a 5 mg ml$^{-1}$ MTT in phosphate buffered saline (PBS) solution was added to each well. Plates were then centrifuged (30 min, 150 *g*). The supernatant was aspirated, taking special care not to disturb the formazan crystals. Dimethyl sulphoxide (100%, 200 μl) was used to solubilise the formazan crystals and the plate was read immediately at 520 nm using a scanning microtitre well spectrophotometer (Flow Lab, Irvine, Scotland). Surviving fraction was calculated by the equation mean of test sample/mean of untreated sample. Controls consisted of media without cells (background extinction), and cells incubated in microculture wells with medium without the drug. Four experiments were performed in triplicate.

### *Trypan blue exclusion test*

Cell survival, until 22 h after CDDP exposure, was checked with trypan blue exclusion (0.4% trypan blue solution in PBS diluted 1:1 with a cellular suspension).

### *CDDP treatment for the study of Pt-DNA effects*

Cells ($5 \times 10^7$) were treated with 100 μM CDDP in 50 ml medium (RPMI with 10% FCS). Cells were harvested for

Correspondence: N.H. Mulder.
Received 7 June 1989; and in revised form 28 July 1989.

Pt-DNA binding experiments by AAS and the immunochemical quantitation of Pt-DNA adducts in DNA digested samples during the CDDP treatment (at 1 and 2 h) and after the 2 h CDDP treatment at 4, 10 and 22 h.

After a 2 h CDDP treatment, cells were washed twice with PBS (37°C) and resuspended for further culture in fresh medium at 37°C. For the repair period $t = 0$ h the cells were resuspended in fresh medium at 0°C. After a repair period ($t = 0$ h, $t = 4$ h, $t = 22$ h) the cells were washed twice with PBS (0°C), pelleted by centrifugation and frozen. For the 1 h CDDP treated cells, the cells were washed twice with PBS (0°C), once with fresh medium (0°C and again twice with PBS (0°C), pelleted by centrifugation and frozen (−20°C). These five washings in the 1 h CDDP treated cells were performed in order to be able to compare the results of the 1 and 2 h CDDP treatments. Two separate experiments were performed.

*DNA synthesis*

The DNA synthesis was measured in $GLC_4$ and $GLC_4$-CDDP after a 2 h 100 μM CDDP exposure as described by Bedford *et al.* (1988), with minor modifications.

Briefly, cells were labelled for 24 h with 0.20 μCi $ml^{-1}$ $^3$H-thymidine followed by 4 h in isotope free medium. Cells were exposed for 2 h to 100 μM CDDP and harvested at 0, 4, 10 and 22 h. DNA was extracted by heating cell pellets at 70°C for 1 h in 1 N perchloric acid. The $^3$H radioactivity was determined by scintillation counting and the DNA content was estimated spectrophotometrically at 260 nm. The dilution factor was calculated by dividing the specific activity of DNA at 4, 10 or 22 h by the specific activity of DNA at 0 h. Two experiments were performed in duplicate.

*DNA isolation*

The DNA from frozen pellets ($5 \times 10^7$ cells) was isolated as described by Fichtinger-Schepman *et al.* (1987). Briefly, a phenol extraction and ethanol precipitation was followed by a RNase treatment. The remaining proteins were extracted by chloroform/isoamylalcohol.

*Quantitation of total Pt bound to DNA*

After DNA isolation ($5 \times 10^7$ cells) the DNA was solubilised in 1 M HCl. The DNA content was measured spectrophotometrically at 260 nm (extinction of 1 mg DNA $ml^{-1} = 27$). The Pt content was determined by ASS (Varian Techtron Pty Ltd, Mulgrave, Victoria, Australia) (Hospers *et al.*, 1988). The two different experiments were performed each in duplicate.

*Immunochemical quantitation of Pt-DNA adducts*

The Pt-GG, Pt-AG, Pt-GMP and G-Pt-G adducts were measured using three different polyclonal antibodies according to Fichtinger-Schepman *et al.* (1987). After DNA isolation and digestion, the DNA products were separated on the Mono Q column (Pharmacia, Sweden). After preparing a standard curve with DNA of the cells, the total DNA content was determined from the dGMP peak height of the high performance liquid chromatography pattern. The content of the different adducts in the eluate fraction was determined in a competitive enzyme linked immunosorbent assay (ELISA). The two different experiments were each performed in three different competitive ELISAs and each ELISA was performed in four dilutions in duplicate.

*Statistical analysis*

Differences were tested using the paired and unpaired Student's $t$ test with $P < 0.05$ considered as significant.

Error bars in Figures 1–3 are given for cumulative data in repeat experiments.

### Results

*Survival*

No cell loss or loss in viability for both cell lines, as tested with the trypan blue assay, was detected 22 h after CDDP treatment in the concentrations used.

Figure 1 shows the dose–response curves for CDDP treatment in $GLC_4$ and $GLC_4$-CDDP as tested in the MTA assay. $GLC_4$-CDDP had a RF of 11 based on the $IC_{50}$, the dose inhibiting cell survival by 50%. Thirty per cent of $GLC_4$-CDDP cells survived 72 h after a 2 h exposure to 100 μM CDDP.

*Formation and removal of CDDP induced Pt-DNA adducts*

During the period after the end of the CDDP treatment, a decrease in the adduct content per DNA amount could be due to either removal or DNA synthesis. The aim of this study was to measure the removal and therefore all presented data are corrected for DNA synthesis. The dilution factor for $GLC_4$ at 4, 10 and 22 h post-CDDP treatment was 0.93 ± 0.03 (s.e.), 0.89 ± 0.02 and 0.83 ± 0.03, respectively. The dilution factor for $GLC_4$-CDDP at 4, 10 and 22 h post-CDDP treatment was 0.94 ± 0.02, 0.91 ± 0.02 and 0.86 ± 0.01, respectively.

Figure 2 shows the Pt-DNA adduct formation and removal in $GLC_4$ and $GLC_4$-CDDP. During the last hour of the CDDP treatment, there was an equal net Pt-DNA formation rate. The net formation was higher at the end of the incubation in $GLC_4$, suggesting a more rapid net platination at the beginning. In the post-treatment period there was a significant repair from 0 to 4 h in the $GLC_4$-CDDP (approximately 20% reduction) and no repair in $GLC_4$. After 4 h, no further reduction of Pt-DNA was seen in $GLC_4$-CDDP.

Figure 3 shows the Pt-GG, Pt-AG, G-Pt-G and Pt-GMP adduct formation and removal in $GLC_4$ and $GLC_4$-CDDP. There was a higher net Pt-GG formation rate during CDDP treatment in $GLC_4$. While there was a significant removal over a 4 h period (approximately 30% reduction) seen in $GLC_4$, no removal was seen in $GLC_4$-CDDP. The Pt-AG adduct formation was, in contrast to other adducts, formed to a large degree after exposure to CDDP has been completed. Pt-AG adduct formation was slow. Its maximum was reached after 10 h post-treatment. After 10 h there was significant Pt-AG repair in both cell lines. The G-Pt-G adduct showed a slower net adduct formation rate in $GLC_4$-CDDP. No significant repair was found for both cell lines.

**Figure 1** Cytotoxicity with MTA after 2 h incubation with CDDP with $GLC_4$ (O–O) and $GLC_4$-CDDP (●–●). Bars s.e. ($n$ = 7–12). Statistics $GLC_4$ versus $GLC_4$-CDDP: D, $P < 0.0005$.

**Figure 2** Pt-DNA binding after 1 and 2 h 100 µM CDDP treatment. After 2 h 100 µM CDDP treatment the Pt-DNA binding, in $GLC_4$ (O–O) and $GLC_4$-CDDP (●–●), is given as a function of length of a post-treatment incubation period. Bars s.e. ($n = 4$). Statistics $GLC)_4$ versus $GLC_4$-CDDP: D, $P < 0.0005$. (●–●) decrease (C).

The Pt-GMP adduct showed a significant difference in formation after the first h CDDP treatment. No difference was found after 2 h CDDP treatment. A significant but equal Pt-GMP adduct removal was found in both cell lines.

The distribution of the adducts is shown in Table I. The major adduct was the Pt-GG in both cell lines. There was an increased percentage of the Pt-GG adducts in $GLC_4$ at each time point and a decreased percentage of the Pt-AG and G-Pt-G adducts as compared with $GLC_4$-CDDP.

**Table I** Distribution of adducts as a percentage of total platination in $GLC_4$ and $GLC_4$-CDDP after 1 and 2 h 100 µM CDDP treatment, and 10 and 22 following the 2 h 100 µM CDDP exposure

| | *$GLC_4$* | *$GLC_4$-CDDP* |
|---|---|---|
| | *Amount as % of total platination* | |
| *Pt-GG* | | |
| − 1 h | 69 | 48 |
| 0 h | 79 | 51 |
| 10 h | 61 | 58 |
| 22 h | 75 | 60 |
| *Pt-AG* | | |
| − 1 h | 19 | 33 |
| 0 h | 10 | 23 |
| 10 h | 27 | 28 |
| 22 h | 15 | 26 |
| *G-Pt-G* | | |
| − 1 h | 5 | 13 |
| 0 h | 5 | 12 |
| 10 h | 8 | 10 |
| 22 h | 9 | 12 |
| *Pt-GMP* | | |
| − 1 h | 7 | 5 |
| 0 h | 6 | 15 |
| 10 h | 4 | n.d. |
| 22 h | 2 | 2 |
| | *Amount (fmol Pt µg⁻¹ DNA) ± s.e.* | |
| *Total*[a] | | |
| − 1 h | 54 ± 3.4 | 33 ± 4.6 |
| 0 h | 282 ± 30 | 102 ± 11 |
| 10 h | 244 ± 17 | 148 ± 21 |
| 22 h | 214 ± 19 | 85 ± 20 |

[a]Total binding was calculated by adding together the amounts of the four individual adducts measured by the immunochemical method.

### Discussion

The spectrum of adducts found between CDDP and DNA (Pt-GG, Pt-AG, Pt-GMP and G-Pt-G) does not differ between isolated DNA and various cell types (Fichtinger-Schepman *et al.*, 1985, 1987, 1988; Bedford *et al.*, 1988; Plooy *et al.*, 1985). However, quantitative differences exist between adducts found in different cell lines with widely different sensitivity for cisplatin (Fichtinger-Schepman *et al.*, 1988). Therefore, the study of the kinetics of adduct formation could be relevant for the problem of CDDP resistance. Eastman *et al.* (1988) found a relationship between the Pt-GG adduct repair and the RF in murine leukaemia L1210 cells. These Pt-GG adducts are induced by (3H)-*cis*-dichloro(ethylene-diamine)platinum (II) (*cis*-DEP), a CDDP analogue. In this experiment, we studied the formation and

**Figure 3** Pt-GG, Pt-AG, G-Pt-G and Pt-GMP adduct content after 1 h and 2 h 100 µM CDDP treatment. After 2 h 100 µM CDDP treatment the Pt-GG, Pt-Ag, G-Pt-G and Pt-GMP adduct content, in $GLC_4$ (O–O) and $GLC_4$-CDDP (●–●) is given as a function of length of a post-treatment incubation period. Bars s.e. ($n$ = 3–6). Statistics $GLC_4$ versus $GLC_4$-CDDP: B, $P < 0.05$; C, $P < 0.005$; D, $P < 0.0005$. Pt-GG: O–O decrease (C). Pt-AG: O–O/●–● $t$ = 10 h increase (B). G-Pt-G: O–O/●–● increase − 1 to 0 h (C). Pt-GMP: O–O/●–● increase − 1 to 0 h (C), O–O/●–● decrease 0 to 4 h (D).

repair of the CDDP induced DNA adducts: Pt-GG, Pt-AG, Pt-GMP and G-Pt-G, in a CDDP resistant and sensitive human SCLC cell line with the help of polyclonal antibodies.

In a previous experiment on the platination of $GLC_4$ and its CDDP resistant subline $GLC_4$-CDDP (Hospers *et al.*, 1988) we described a difference in formation of interstrand cross-links, but not in Pt-GG adducts as is found in the experiments described here. This difference, though possibly influenced by a somewhat higher CDDP concentration used, 100 $\mu$M now versus 67 $\mu$M maximum previously, may more probably be due to a more important factor, that being the substantial increase in resistance (RF 11 versus 6.4). These observations underline the importance of the degree of resistance in studies on resistance mechanisms.

Adding to our previous observations of differences in the formation of total platination and interstrand cross-links between sensitive and resistant cells, we now also detect differences in Pt-GG and Pt-AG adduct formation. Total platination occurs more rapidly in the sensitive line, and total Pt-GG adduct formation is 68% higher at the end of the CDDP exposition.

The difference in the quantitatively important Pt-GG adducts is more likely to correspond to resistance than the difference in the formation of Pt-AG adducts. Although the Pt-AG adducts can be cytotoxic, or at least mutagenic (Burnouf *et al.*, 1987) a form of repair equalises the differences in sensitive and resistant cells as far as Pt-AG adducts are concerned. It is not clear why these adducts can be formed in the hours after exposition. Their number is too high to be explained by monoadduct conversion only. They may be the result of residual free CDDP, but no difference in cellular and nuclear Pt concentration was found (Hospers *et al.*, 1988) to explain the differences in kinetics.

This lack of differences in Pt concentrations also suggests that differences in the net formation of Pt-DNA adducts between both cell lines is most likely due either to differences in repair of adducts or to differences at the DNA level; for instance, in the conformational state of the DNA. As far as the Pt-GG adducts are concerned, the lack of evidence for their repair in the $GLC_4$-CDDP cell line favours the role of a DNA factor. Repair does seem to be present in $GLC_4$-CDDP as far as the total platination is concerned (Figure 2). From the adducts measured in this study with the immunochemical method none can explain this decrease in platination during the first 4 h after exposition as measured by the AAS (Figure 2), especially in view of the minute quantitative role of adducts other than Pt-GG. We suggest that some adduct is formed in the resistant cell that cannot be detected with the immunochemical method used, and that is repairable and less toxic to the cell than, for instance, the Pt-GG adduct. Formation of such an adduct might prevent the production of more toxic adducts; in combination with differences in cell cycle characteristics this might explain the resistance.

Speculations on the nature of this adduct might be directed by the finding of an elevated level (2.5-fold) of total GSH in $GLC_4$-CDDP (Hospers *et al.*, 1988). This might give rise to the formation of a GSH-Pt-DNA adduct as described by Eastman (1987).

Additional experiments are necessary to analyse further these differences in adduct formation and their possible role in resistance.

The expertise of A.M.J. Fichtinger-Schepman, Medical Biological Laboratory TNO Rijswijk, The Netherlands, is gratefully acknowledged. Supported in part by a grant from the Dutch Cancer Foundation, GUKC 86-1.

### References

BEDFORD, P., FICHTINGER-SCHEPMAN, A.M.J., SHELLARD, S.A., WALKER, M.C., MASTERS, J.R.W. & HILL, B.T. (1988). Differential repair of platinum-DNA adducts in human bladder and testicular tumor continuous cells lines. *Cancer Res.*, **48**, 3019.

BURNOUF, D., DAUNE, M. & FUCHS, R.P.P. (1987). Spectrum of cisplatin-induced mutations in *Escherichia coli*. *Proc. Natl Acad. Sci. USA*, **84**, 3758.

CARMICHAEL, J., DE GRAFT, W.G., GAZDAR, A.F., MINNA, J.D. & MITCHELL, J.B. (1987). Evaluation of tetrazolium-based semiautomated colorimetric assay: assessment of chemosensitivity testing. *Cancer Res.*, **47**, 936.

EASTMAN, A. (1987). Cross-linking of glutathione to DNA by cancer chemotherapeutic platinum coordination complexes. *Chem. Biol. Interactions*, **61**, 241.

EASTMAN, A., SCHULTE, N., SHEIBANI, N. & SORENSON, C.M. (1988). Mechanisms of resistance to platinum drugs. In *Platinum and Other Metal Coordination Compounds in Cancer Chemotherapy*, Nicolini, M. (ed.) p. 178. Martinus Nijhoff: Boston.

FICHTINGER-SCHEPMAN, A.M.J., VAN DER VEEN, J.L., DEN HARTOG, J.H.J., LOHMAN, P.H.M. & REEDIJK, J. (1985). Adducts of the antitumor drug *cis*-diammine-dichloroplatinum (II) with DNA: formation, identification, and quantitation. *Biochemistry*, **24**, 707.

FICHTINGER-SCHEPMAN, A.M.J., VAN OOSTEROM, A.T., LOHMAN, P.H.M. & BERENDS, F. (1987). Cisplatin-induced DNA adducts in peripheral leucocytes from seven cancer patients: quantitative immunochemical detection of the adduct induction and removal after a single dose of cisplatin. *Cancer Res.*, **47**, 3000.

FICHTINGER-SCHEPMAN, A.M.J., DIJT, F.J., DE JONG, W.H., VAN OOSTEROM, A.T. & BERENDS, F. (1988). *In vivo cis*-diamminedichloroplatinum (II)-DNA adduct formation and removal as measured with immunochemical techniques. In *Platinum and Other Metal Coordination Compounds in Cancer Chemotherapy*, Nicolini, M. (ed.) p. 32. Martinus Nijhoff: Boston.

HOSPERS, G.A.P., MULDER, N.H., DE JONG, B. & 4 others (1988). Characterization of a human small cell lung carcinoma cell line with acquired resistance to *cis*-diamminodichloroplatinum (II) *in vitro*. *Cancer Res.*, **48**, 6803.

PLOOY, A.C.M., FICHTINGER-SCHEPMAN, A.M.J., SCHUTTE, H.H., VAN DIJK, M. & LOHMAN, P.H.M. (1985). The quantitative detection of various Pt-DNA adducts in chinese hamster ovary cells treated with cisplatin: application of immunochemical techniques. *Carcinogenesis*, **6**, 561.

ROBERTS, J.J., KNOX, R.J., FRIEDLOS, F. & LYDALL, D.A. (1986). DNA as the target for the cytotoxic and anti-tumour action of platinum co-ordination complexes: comparative *in vitro* and *in vivo* studies of cisplatin and carboplatin. In *Biochemical Mechanisms of Platinum Antitumour Drugs*, McBrien, D.C.H. & Slater, T.F. (eds) p. 29. IRL Press: Oxford.

SCANLON, K.J., SAFIRSTEIN, R.L., THIES, H., GROSS, R.B., WAXMAN, S. & GUTTENPLAN, J.B. (1983). Inhibition of amino acid transport by *cis*-diamminedichloro-platinum (II) derivates in L1210 murine leukemia cells. *Cancer Res.*, **43**, 4211.